# Preoperative risk factors for suboptimal initial clinical response or weight regain in patients undergoing bariatric surgery, a retrospective cohort study from a high-volume center

**DOI:** 10.1007/s00423-025-03700-0

**Published:** 2025-04-22

**Authors:** Enrique Salazar-Rios, Cesar A. Martínez Ortíz, Maria E. Salazar-Rios, Carlos A. Gutiérrez Rojas

**Affiliations:** 1 División de Estudios de Posgrado, Facultad de Medicina, Universidad Nacional Autónoma de México, Mexico City, México; 2 Departamento de Gastrocirugía, Hospital de Especialidades “Dr. Bernardo Sepúlveda Gutiérrez” Centro Médico Nacional Siglo XXI, Instituto Mexicano del Seguro Social, Mexico City, Mexico; 3 Hospital de Pediatría UMAE “Dr. Silvestre Frenk Freund” Centro Médico Nacional Siglo XXI, Instituto Mexicano del Seguro Social, Mexico City, Mexico

**Keywords:** Bariatric surgery, Insufficient weigh loss, Surgical outcomes, Predictive factors

## Abstract

**Introduction:**

Bariatric surgery is widely recognized as a mainstay in the treatment of obesity; however, there is limited information regarding its success and the factors that influence outcomes within the Mexican population. This study provides an analysis of bariatric surgery outcomes at the “Hospital de Especialidades” of the “Centro Médico Nacional Siglo XXI,” with a particular focus on the prevalence of suboptimal initial clinical response, weight regain, and the identification of predictive factors.

**Methods:**

A retrospective cohort study involving 132 patients who underwent bariatric surgery between January 2018 and March 2023 was conducted. The prevalence of suboptimal initial clinical response was determined, and a binary logistic regression was applied to identify potential risk factors.

**Results:**

The study found that 21.97% of patients experienced suboptimal initial clinical response, a rate lower than reported in global literature. The population exhibited a significant prevalence of comorbidities, including type 2 diabetes mellitus (63.64%), hypertension (55.3%), and obstructive sleep apnea (60.61%), reflecting Mexico’s high obesity rates. Additionally, male sex was identified as a significant predictor of suboptimal initial clinical response, while glycated hemoglobin and serum albumin emerged as relevant biochemical predictors, underscoring the importance of preoperative glycemic control.

**Conclusion:**

These findings offer valuable insights into bariatric surgery outcomes and identifies adequate preoperative glycemic control as an important modifiable factor that can inform future policies aimed at enhancing patient care and surgical success in bariatric procedures.

**Supplementary information:**

The online version contains supplementary material available at 10.1007/s00423-025-03700-0.

## Introduction

Obesity is a complex disease with a significant impact on modern society, and its prevalence has increased markedly over the last 50 years [1]. The World Health Organization defines obesity as an excessive accumulation of body fat that adversely affects health, characterized by a body mass index (BMI) greater than 30 kg/m^2^.[2] Obesity constitutes a major public health issue that reduces life expectancy and is a leading cause of preventable death worldwide. [2] Additionally, it increases the risk of numerous health conditions such as type 2 diabetes, cardiovascular diseases, and metabolic syndrome, among others [3]. In Mexico, the situation is equally alarming. According to the 2022 National Health and Nutrition Survey, 41.0% of adult women and 32.3% of adult men were classified as obese, highlighting the significant impact of obesity on public health [4].

Surgical treatment of obesity has proven more effective over time, with notable impact on controlling cardiovascular diseases common in this population [5, 6]. These patients typically experience a significant reduction in BMI of approximately 12 to 17 points, comparable to about 45–70% of excess weight loss. Moreover, there is a marked improvement in obesity-related comorbidities [7–9]. Therefore, bariatric surgery is considered a cornerstone in the treatment of obesity.

The percentage of excess weight loss (%EWL) is commonly used to evaluate the success of bariatric surgery [10], and while most patients undergoing bariatric surgery achieve successful and sustained weight loss, approximately 15–20% of patients do not experience a favorable outcome due to suboptimal initial clinical response or long-term weight regain[2, 11, 12] and up to 30% of patients who undergo bariatric procedures may not reach an adequate postoperative %EWL [13, 14]. The most widely accepted definition for suboptimal initial clinical response is an %EWL of less than 50% after 12 months, while weight regain is defined as an increase in %EWL greater than 25% of the minimum weight achieved [15]. The optimal postoperative time frame to evaluate these conditions also remains a subject of debate, with many studies showing that peak weight loss occurs between one and three years after surgery [15]. Some studies have observed a weight regain of 3.9% at three years after Roux-en-Y gastric bypass, with estimates suggesting this number could be as high as 10–20% in the long term [2, 16].

Incomplete response to surgery is a leading cause for revision procedures, which increases patient morbidity and imposes a significant financial burden on hospitals. Additionally, it has been linked to an increased recurrence of metabolic comorbidities [17]. Thus, identifying preoperative factors that can predict suboptimal initial clinical response has become crucial. Previous studies have evaluated demographic characteristics, surgical factors, biochemical markers and coexisting pathological conditions, however, results have been conflicting [18, 19]. Within Mexico, there is limited information regarding rates of suboptimal initial clinical response, and a lack of studies addressing the factors influencing the success of these procedures. Given the variability in surgical outcomes, this study aims to find the prevalence of indaqueate response to bariatric surgery, and identify preoperative factors that may predict suboptimal initial clinical response in patients undergoing bariatric surgery. Due to the high incidence of comorbidities in our country, we hypothesize that demographic, biochemical, and pathological variables—including sex, glycated hemoglobin, albumin, and comorbidities such as T2DM, HTN, and OSA—are associated with an increased risk of inadequate weight loss or weight regain following surgery.

## Materials and methods

A retrospective cohort was conducted on patients who underwent bariatric surgery at the Obesity Clinic of the"Hospital de Especialidades"at the “Centro Médico Nacional Siglo XXI” of the Mexican Social Security Institute between January 2018 and March 2023. The study included patients over 18 years old with a BMI over 40 kg/m^2^ or over 35 kg/m^2^ with a comorbidity, and at least one year of follow-up. Patients with incomplete records or lost during follow-up were excluded. The study followed the 1964 Helsinki declaration and complied with national legislation on health research. Additionally, it was approved by the local ethics committee (R- 2024–3601 - 028), with formal consent not required due to minimal risk.

Demographic, anthropometric, and clinical data (including comorbidities and type of procedure) were collected for each patient, along with preoperative laboratory results. Post-surgery, anthropometric data were recorded at 1, 3, 6, and 12 months. The BMI of each patient was calculated using the formula: $BMI=\frac{weight (kg)}{{height (m)}^{2}}$. The %EWL was calculated considering the preoperative BMI and the BMI recorded at each follow-up visit using the following formula: $\%EWL=\frac{initial BMI-current BMI}{initial BMI-ideal BMI}*100$. An ideal BMI of 25 kg/m^2^ was used as a reference. Suboptimal initial clinical response was defined as %EWL < 50% at 12 months, and weight regain as an increase in %EWL > 25% from the minimum weight achieved after surgery.

### Statistical analysis

A Binary Logistic Regression (BLR) model was used to identify predictors of suboptimal initial clinical response, incorporating demographic (sex, height, weight, BMI, age), biochemical (hemoglobin, glycated hemoglobin, albumin, glucose, and proteins), and pathological variables (Type 2 Diabetes Mellitus (T2DM), hypertension (HTN), and obstructive sleep apnea (OSA)). The model, $\text{P}\left(\text{Y}\right)=\frac{1}{1+{\text{e}}^{-\left(\text{a}+\text{bX}\right)}}$ predicted surgery outcomes (coded as 0 for suboptimal response, 1 for success) based on independent variables. A stepwise backward elimination method removed insignificant covariates, with model significance tested via Chi-square and Wald statistics. Receiver operating characteristic (ROC) curve analysis was performed on cuantitative variables considered significant. Chi-square contingency tables were used to test the association between surgery outcomes and predictors. The null hypothesis (H_0_) proposed no significant association, while the alternative hypothesis (H_A_) suggested a significant association with p < 0.05 considered significant.

## Results

Between January 2018 and March 2023, 182 patients were treated at the Obesity Clinic. Of these, 26 patients with surgical conversions, 2 with anatomy reversals, 1 lacking preoperative data, 2 operated elsewhere, and 19 without at least one year of follow-up were excluded. The study included 132 patients, with demographic, biochemical, and pathological data shown in Table 1. Most patients were women (75.76%), with an average age of 44.38 years, height of 1.62 m, weight of 117.75 kg, and BMI of 44.9 kg/m^2^. Biochemical data revealed average glucose of 106.14 mg/dL, glycated hemoglobin of 5.98 g/dL, hemoglobin of 14.45 g/dL, albumin of 4.19 g/dL, and total proteins of 6.91 g/dL. Comorbidities included T2DM (63.64%), HTN (55.30%), and OSA (60.61%). Surgical procedures performed were Roux-en-Y gastric bypass (RYGB) (n. 57, 43.18%), one-anastomosis gastric bypass (OAGB) (n. 45, 34.09%), and sleeve gastrectomy SG (n. 30, 22.73%). After one year follow-up, 21.97% of patients exhibited suboptimal initial clinical response, none of our patients met the definition of weight regain.

**Table 1:** Demographic, biochemical and pathologic characteristics analyzed in the Cohort

	Variable	n	Female %	Male %	
Demographic	Sex	132	75.76	24.24	
	Variable	n	Mean ± C.I. 95%	Maximum	Minimum
	Age (years)	132	44.38 ± 1.58	77	20
	Height (m)	132	1.62 ± 3.76	1.79	1.46
	Weight (Kg)	132	117.75 ± 1.25	201.50	78.00
	BMI (kg/m^2^)	132	44.98 ± 1.26	33.76	70.67
Biochemical	Variable	n	Mean ± C.I. 95%	Maximum	Minimum
	Glucose (mg/dl)	132	106.14 ± 8.59	579.00	69.00
	HbA1c % (g/dL)	112	5.98 ± 0.24	11.90	4.70
	Hemoglobin (g/dL)	132	14.45 ± 0.25	18.50	10.60
	Albumin (g/dL)	125	4.19 ± 0.07	5.90	3.00
	Proteins (g/dL)	125	6.91 ± 0.12	8.30	3.50
Pathologic	Variable	n	Present %	Absent %	
	T2DM	132	63.64	36.36	
	HTN	132	55.30	44.70	
	OSA	132	60.61	39.39	

### Demographic variables

**Fig. 1:**
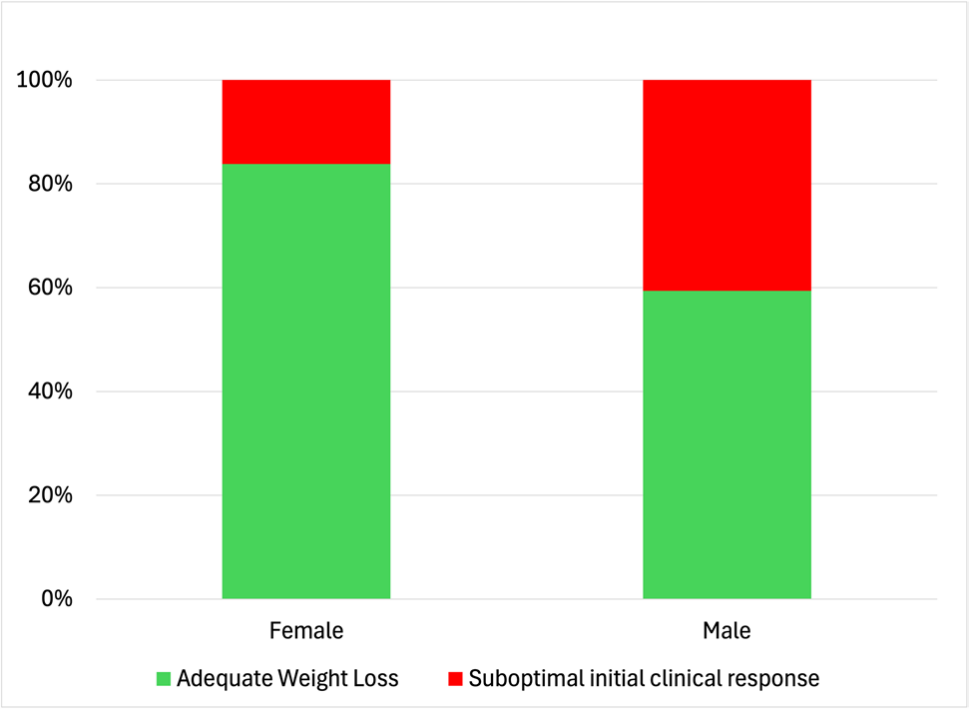
Frequency distribution of outcomes in bariatric surgery concerning sex

The BLR for demographic variables resulted in a model with 77.8% accuracy and a constant value of − 1.253 (Table S1), with a Chi-square value of 4.244 (1 degree of freedom) and a significance of 0.039. Nagelkerke’s R^2^ indicated that the model at step 5 explained 5.90% of the variation in surgical outcomes (Table S2). Using the Wald statistic (Table S3), height (0.167) was removed in step 2, age (0.449) in step 3, BMI (0.409) in step 4, and degree of obesity in step 5. Only sex was found to be a significant predictor of surgical outcomes, with males being more likely to have a suboptimal initial clinical response. (Fig. 1).

### Biochemical variables

In the biochemical variables analysis, the model’s accuracy was 81.1% when only the constant was included, with a constant value of − 1.229 (Table S4). A Chi-square value of 16.809 (4 degrees of freedom) and significance of 0.002 indicated significant differences. In step 2, Nagelkerke R^2^ explained 22.3% of the dependent variable change, suggesting biochemical variables are moderately predictive (Table S5). Stepwise analysis removed glucose in step 2 (0.100). Glycated hemoglobin and total serum albumin were identified as significant predictors, with Wald values of 4.039 (p = 0.044) and 5.199 (p = 0.023), respectively. Total serum proteins (Wald = 3.605, p = 0.058) did not reach statistical significance but showed a potential association.

**Fig. 2:**
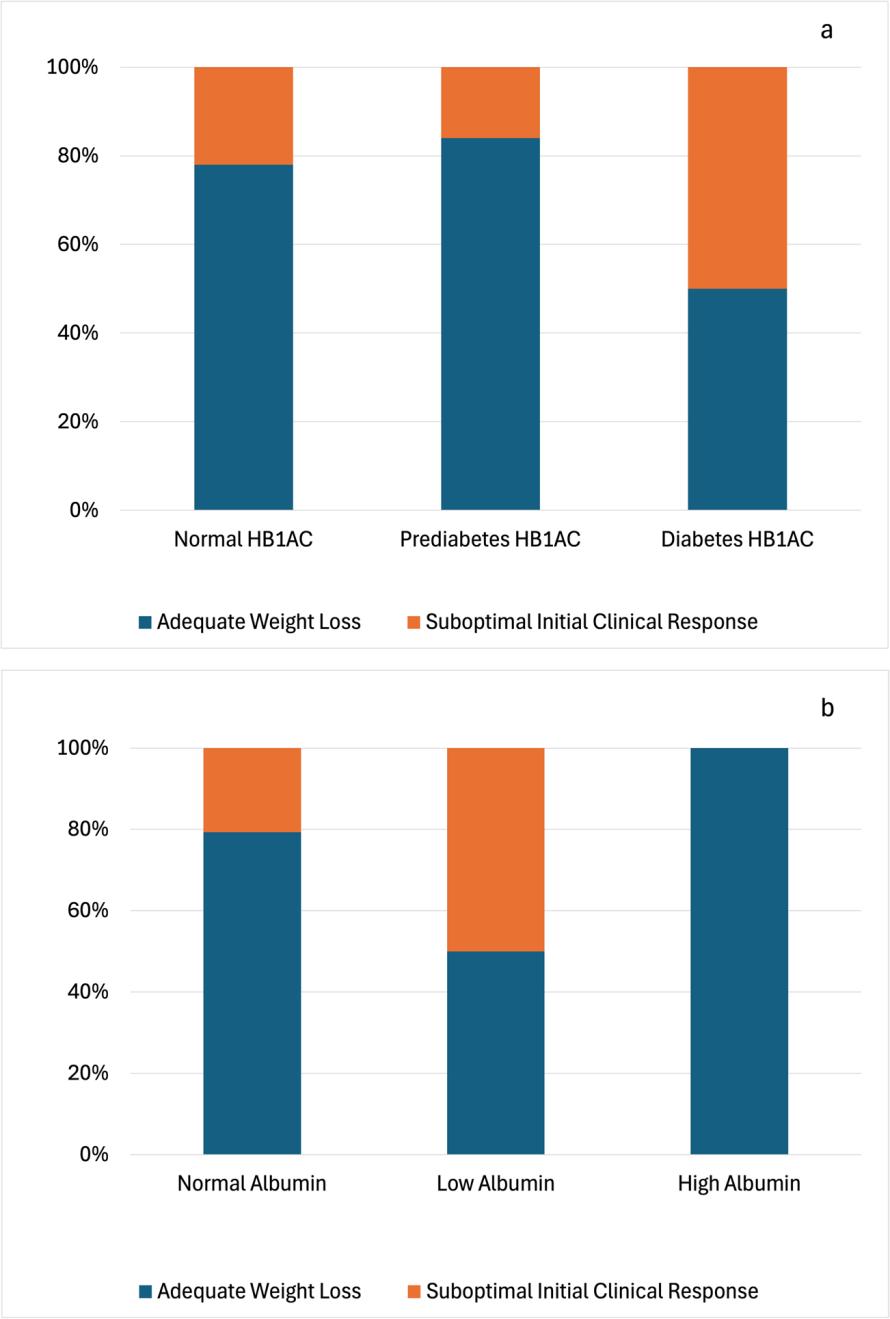
Frequency distribution of outcomes in bariatric surgery concerning biochemical aspects. a: Glycated hemoglobin; b: Serum albumin

ROC curve analysis confirmed that glycated hemoglobin, and albumin are statistically significant predictors, however, they exhibit low specificity (22.7%, 18.9%, and 17.8% respectively) and moderate sensitivity (37.0%, 31.8%, and 28.4% respectively). The comparison of reference ranges revealed several findings (Figs. 2a- 2b). Glycated hemoglobin (Fig. 2a) showed higher rates of suboptimal initial clinical response in patients with values above the acceptable range. Meanwhile, patients with lower albumin levels exhibited higher rates of suboptimal initial clinical response (Fig. 2b).

### Pathological variables

In the pathological variables analysis, an accuracy rate of 77.8% and a constant value of − 1.253 were observed (Table S7). A Chi-square value of 3.748 (1 degree of freedom, p = 0.053) and Nagelkerke R^2^ in step 4 showed no significant differences when including pathological variables (Table S8). The degree of obesity, with a significance of 0.095, was the closest predictor but was not statistically significant (Table S9).

## Discussion

Bariatric surgery is a key treatment for obesity but lacks outcome data and influencing factors in our country. As obesity becomes more prevalent, optimizing surgical treatment is essential. This retrospective analysis of patients undergoing bariatric surgery at the Obesity Clinic aimed to assess procedure effectiveness and the prevalence of suboptimal initial clinical response and weight regain. Our clinic’s most common procedure is laparoscopic RYGB (43.18%), followed by OAGB (34.09%) and SG (22.73%), differing from global trends where SG is the most common, performed in nearly 50% of cases[20, 21].

The prevalence of T2DM (63.64%), HTN (55.3%), and OSA (60.61%) in our population is notably higher than global averages (19.8%, 30.6%, and 18.6%, respectively) [20]. Given the high obesity rates in Mexico, these elevated numbers are expected, highlighting the urgent need to address obesity as a public health issue. Our data shows that 21.97% of patients had a suboptimal initial clinical response after bariatric surgery, lower than the literature average of 30% [13, 14]. Our center is unique in serving the entire bariatric patient population covered by Mexico’s largest public health institution. As a high-volume center for this condition, we anticipate higher success rates. Additionally, despite global trends favoring SG, our center performs RYGB more frequently, which may contribute to the lower suboptimal response rate, as RYGB has a slight advantage in %EWL and long-term weight regain [22].

In our cohort, no patients met the definition of weight regain, likely due to the short follow-up period. The literature provides conflicting data on the optimal timing for evaluating the success of bariatric surgery. Bariatric surgery’s maximum effects are typically observed 12 months post-intervention, potentially extending up to 3 years [15]. Weight regain often starts 2 years after reaching minimum weight and can continue for up to 5 year [23]. Unfortunately, a limitation in our study was the follow-up time for patients. Often, after the first year of follow-up, patients did not continue with subsequent appointments, making long-term evaluation impossible. Additionally, the SARS-CoV- 2 pandemic disrupted the follow-up for several patients who, despite undergoing bariatric procedures, missed subsequent appointments or even passed away, leaving their outcomes uncertain. Thus, future studies could benefit from extended follow-up to identify cases of weight regain. Additionally, extending and ensuring follow-up for these patients at the Obesity Clinic could provide better insight into the long-term outcomes of bariatric surgery and help improve obesity management and treatment strategies.

Among the demographic factors analyzed, only sex was statistically significant, with men being more likely to experience a suboptimal initial clinical response compared to women. This finding is consistent with existing literature, suggesting that this segment of the population should be specifically targeted for other interventions that promote adequate weight loss, such as psychological treatment for eating disorders, as well as diet and exercise.[24, 25] In our cohort, preoperative BMI did not reach statistical significance as a predictive factor. However, previous studies have linked higher pre-surgical BMI with greater failure rates suggesting that a lower initial weight improves surgical outcomes [13]. Therefore, preoperative diet and exercise plans should be emphasized to enhance surgical success.

Among biochemical factors, glycated hemoglobin was found as a significant predictor for bariatric surgery outcomes, with higher suboptimal initial clinical response rates observed in patients with values exceeding the reference limit. This is consistent with literature, which suggests that diabetic patients with elevated glycated hemoglobin levels experience less weight loss after surgery [26, 27]. Additionally, lower albumin levels also showed a significant association with suboptimal initial clinical response. This agrees with previous literature where lower albumin levels have been associated with inadequate weight loss, and a greater incidence of postoperative complications [28, 29]. The management and control of biochemical parameters in patients, especially those with obesity who experience significant changes due to comorbidities, are crucial for improving outcomes, as shown in our results. Thus, during preoperative evaluation, special attention should be given to glycated hemoglobin and serum albumin, and efforts to correct any deviations should be prioritized before undergoing surgery.

Of the pathological variables analyzed, none reached statistical significance as risk factors, with obesity degree being the closest to this. Only hypertension showed a mild association with suboptimal initial clinical response. Patients with T2DM or OSA had similar success rates to those without these conditions. Literature shows a link between T2DM and suboptimal initial clinical response[30] while results for OSA are mixed [13, 14, 25]. Despite these findings, it is important to recognize that comorbidities, especially when multiple conditions are present, can increase the risk of postoperative complications, which can negatively impact weight loss and overall surgical outcome[31, 32] The presence of these comorbidities, even in the absence of a clear association with the clinical response to surgery, can still contribute to a more challenging recovery process, increased risk of complications, and longer hospital stays. As such, ensuring that these conditions are effectively managed and under control before surgery is crucial.

Our study’s limitations include the short follow-up period and a limited number of patients due to exclusion criteria. Future studies would benefit from a longer follow-up. Additionally, the heterogeneity of preoperative laboratory tests conducted led us to exclude many patients who did not have complete laboratory values, reducing the number of patients that could be included. Thus, standardizing the preoperative laboratory tests could increase the number of patients included in future studies. Our clinic, a referral center for obesity treatment nationwide and part of the “Instituto Mexicano del Seguro Social”, allows us to generalize findings to the Mexican population. Despite the limitations, our results provide insights into the rate of surgical failure for bariatric procedures in Mexico and highlight important predictive factors to consider before surgery, including maintaining adequate preoperative nutritional status and glycemic control, especially in the male population, which has a higher risk of suboptimal initial clinical response. This could potentially lead to the enhancement of policies at this, and other obesity clinics aimed at improving the quality of care provided to patients.

## Conclusion

Limited information exists on bariatric surgery success and influencing factors in the Mexican population. Our study from the Obesity Clinic at the “Hospital de Especialidades, Centro Médico Nacional Siglo XXI”, reports a 21.97% rate of suboptimal initial clinical response, lower than global averages. High prevalence of comorbidities such as type 2 diabetes, hypertension, and obstructive sleep apnea reflects Mexico’s high obesity rates. M ale patients presented with a higher rate of suboptimal initial clinical response, and elevated glycated hemoglobin and low serum albumin were also linked with higher rates of suboptimal initial clinical response. Thus, the optimization of comorbidities, ensuring the maintenance of biochemical parameters within normal values, with a specific emphasis on glycemic control and proper nutrition, especially in male patients, can decrease the risk of suboptimal initial clinical response after bariatric surgery.

## Supplementary information

Below is the link to the electronic supplementary material.

Supplementary file1 (DOCX 48 KB)

## Data Availability

The data generated for this research is not openly available to preserve patient confidentiality. Data is available, through the corresponding author upon reasonable request.
