# Supplementary material for: Preoperative risk factors for suboptimal initial clinical response or weight regain in patients undergoing bariatric surgery, a retrospective cohort study from a high-volume center
Source: Langenbecks Arch Surg. 2025 Apr 22;410(1):136. doi: 10.1007/s00423-025-03700-0 (PMC12011907; doi:10.1007/s00423-025-03700-0)
Supplement: Supplementary file 1 — Supplementary file1 (DOCX 48 KB) [file 423_2025_3700_MOESM1_ESM.docx]

**Table S1.** Results of BLR for Demographic Variables.

|  | Observed | | Predicted | | |
| --- | --- | --- | --- | --- | --- |
|  |  |  | Surgery Outcome | | Correct Percentage |
|  |  |  | Success | Failure |  |
| Step 0 | Surgery Outcome | Success | 84 | 0 | 100.0 |
|  |  | Failure | 24 | 0 | .0 |
|  | Global Percentage | | 77.8 | | |
|  | Constant | | -1.253 |  |  |

**Table S2.** Model summary for Demographic Variables.

| Step | -2 log likelihood | Cox and Snell R² | Nagelkerke R² |
| --- | --- | --- | --- |
| 1 | 106.631 | 0.070 | 0.106 |
| 2 | 106.798 | 0.068 | 0.104 |
| 3 | 107.258 | 0.064 | 0.098 |
| 4 | 107.660 | 0.061 | 0.093 |
| 5 | 110.173 | 0.039 | 0.059 |

**Table S3.** Predictor variable in the model equation for demographic variables.

|  | | B | Standard error | Wald | df | Sig. | Exp(B) |
| --- | --- | --- | --- | --- | --- | --- | --- |
| Step 1 | Patient’s sex (1) | -1.100 | 0.724 | 2.309 | 1 | 0.129 | 0.333 |
|  | Patient’s height (meters) | -1.665 | 4.079 | 0.167 | 1 | 0.683 | 0.189 |
|  | Patient’s age (years) | 0.016 | 0.029 | 0.310 | 1 | 0.578 | 1.016 |
|  | Initial BMI | 0.026 | 0.037 | 0.491 | 1 | 0.484 | 1.026 |
|  | Degree of obesity | 0.740 | 0.870 | 0.725 | 1 | 0.395 | 2.097 |
|  | Constant | -1.825 | 8.131 | 0.050 | 1 | 0.822 | 0.161 |
| Step 2 | Patient’s sex (1) | -0.890 | 0.506 | 3.096 | 1 | 0.078 | 0.411 |
|  | Patient’s age (years) | 0.019 | 0.028 | 0.449 | 1 | 0.503 | 1.019 |
|  | Initial BMI | 0.029 | 0.037 | 0.598 | 1 | 0.440 | 1.029 |
|  | Degree of obesity | 0.767 | 0.872 | 0.775 | 1 | 0.379 | 2.154 |
|  | Constant | -4.973 | 2.690 | 3.419 | 1 | 0.064 | 0.007 |
| Step 3 | Patient’s sex (1) | -0.895 | 0.506 | 3.130 | 1 | 0.077 | 0.409 |
|  | Initial BMI | 0.023 | 0.036 | 0.409 | 1 | 0.523 | 1.023 |
|  | Degree of obesity | 0.867 | 0.861 | 1.014 | 1 | 0.314 | 2.379 |
|  | Constant | -4.167 | 2.413 | 2.981 | 1 | 0.084 | 0.016 |
| Step 4 | Patient’s sex (1) | -0.884 | 0.504 | 3.076 | 1 | 0.079 | 0.413 |
|  | Degree of obesity | 1.097 | 0.779 | 1.982 | 1 | 0.159 | 2.995 |
|  | Constant | -3.755 | 2.336 | 2.583 | 1 | 0.108 | 0.023 |
| Step 5 | **Patient’s sex (1)** | -1.035 | 0.495 | **4.369** | 1 | **0.037** | 0.355 |
|  | Constant | -0.531 | 0.399 | 1.773 | 1 | 0.183 | 0.588 |

**Table S4.** Results of BLR for Biochemical Variables.

|  | Observed | | Predicted | | |
| --- | --- | --- | --- | --- | --- |
|  |  |  | Surgery Outcome | | Correct Percentage |
|  |  |  | Success | Failure |  |
| Step 1 | Surgery Outcome | Success | 80 | 2 | 97.6 |
|  |  | Failure | 19 | 5 | 20.8 |
|  | Global Percentage | | 80.2 | | |
| Step 2 | Surgery Outcome | Success | 80 | 2 | 97.6 |
|  |  | Failure | 2 | 97.6 | 25.0 |
|  | Global Percentage | | 81.1 | | |
|  | Constant | | -1.229 | | |

**Table S5.** Model Summary for Biochemical Variables.

| Step | -2 log likelihood | Cox and Snell R² | Nagelkerke R² |
| --- | --- | --- | --- |
| 1 | 96.481 | 0.148 | 0.225 |
| 2 | 96.592 | 0.147 | 0.223 |

**Table S6.** Predictor variable in the model equation for Biochemical Variables.

|  | | B | Standard error | Wald | df | Sig. | Exp(B) |
| --- | --- | --- | --- | --- | --- | --- | --- |
| Step 1 | Hemoglobin | 0.371 | 0.207 | 3.211 | 1 | 0.073 | 1.449 |
|  | Glycated hemoglobin | 0.322 | 0.241 | 1.779 | 1 | 0.182 | 1.380 |
|  | Serum glucose | 0.002 | 0.007 | 0.100 | 1 | 0.752 | 1.002 |
|  | Serum total albumin | -2.416 | 1.099 | 4.834 | 1 | 0.028 | 0.089 |
|  | Serum total proteins | 1.262 | 0.698 | 3.269 | 1 | 0.071 | 3.534 |
|  | Constant | -7.660 | 4.178 | 3.362 | 1 | 0.067 | 0.000 |
| Step 2 | Hemoglobin | 0.372 | 0.208 | 3.207 | 1 | 0.073 | 1.451 |
|  | **Glycated hemoglobin** | 0.373 | 0.186 | **4.039** | 1 | **0.044** | 1.452 |
|  | **Serum total albumin** | -2.480 | 1.088 | **5.199** | 1 | **0.023** | 0.084 |
|  | Serum total proteins | 1.305 | 0.687 | 3.605 | 1 | 0.058 | 3.689 |
|  | Constant | -7.788 | 4.160 | 3.504 | 1 | 0.061 | 0.000 |

**Table S7**. Results of BLR for Pathological Variables.

|  | Obserrved | | Predicted | | |
| --- | --- | --- | --- | --- | --- |
|  |  |  | Surgery outcome | | Correct percentage |
|  |  |  | Success | Failure |  |
| Step 0 | Surgery outcome | Success | 84 | 0 | 100.0 |
|  |  | Failure | 24 | 0 | .0 |
|  | Global percentage | | 77.8 | | |
|  | Constant | | -1.253 | | |

**Table S8.** Model Summary for Pathological Variables.

| Step | -2 log likelihood | Cox and Snell R² | Nagelkerke R² |
| --- | --- | --- | --- |
| 1 | 111.587 | 0.026 | 0.040 |
| 2 | 111.625 | 0.026 | 0.039 |
| 3 | 111.749 | 0.024 | 0.037 |
| 4 | 114.417 | 0.000 | 0.000 |

**Table S9.** Predictor variable in the model equation for Pathological Variables.

|  | | B | Standard error | Wald | df | Sig. | Exp(B) |
| --- | --- | --- | --- | --- | --- | --- | --- |
| Step 1 | T2DM (1) | 0.176 | 0.522 | 0.113 | 1 | 0.736 | 1.192 |
|  | HTN (1) | 0.631 | 0.523 | 1.454 | 1 | 0.228 | 1.879 |
|  | OSA (1) | -0.138 | 0.496 | 0.077 | 1 | 0.782 | 0.872 |
|  | Degree of obesity | 1.047 | 0.862 | 1.476 | 1 | 0.224 | 2.850 |
|  | Initial BMI | 0.012 | 0.038 | 0.108 | 1 | 0.743 | 1.012 |
|  | Constant | -5.228 | 2.391 | 4.780 | 1 | 0.029 | 0.005 |
| Step 2 | T2DM (1) | 0.189 | 0.520 | 0.133 | 1 | 0.716 | 1.209 |
|  | HTN (1) | 0.620 | 0.521 | 1.416 | 1 | 0.234 | 1.860 |
|  | Degree of obesity | 1.044 | 0.862 | 1.468 | 1 | 0.226 | 2.842 |
|  | Initial BMI | 0.012 | 0.038 | 0.103 | 1 | 0.748 | 1.012 |
|  | Constant | -5.295 | 2.384 | 4.932 | 1 | 0.026 | 0.005 |
| Step 3 | Diabetes mellitus t2(1) | 0.174 | 0.517 | 0.113 | 1 | 0.737 | 1.190 |
|  | HTN (1) | 0.658 | 0.508 | 1.676 | 1 | 0.195 | 1.930 |
|  | Degree of obesity | 1.162 | 0.780 | 2.221 | 1 | 0.136 | 3.196 |
|  | Constant | -5.084 | 2.297 | 4.898 | 1 | 0.027 | 0.006 |
| Step 4 | HTN (1) | 0.662 | 0.508 | 1.697 | 1 | 0.193 | 1.938 |
|  | Degree of obesity | 1.161 | 0.778 | 2.224 | 1 | 0.136 | 3.192 |
|  | Constant | -4.963 | 2.260 | 4.825 | 1 | 0.028 | 0.007 |
| Step 5 | Degree of obesity | 1.281 | 0.768 | 2.780 | 1 | 0.095 | 3.601 |
|  | Constant | -4.894 | 2.240 | 4.773 | 1 | 0.029 | 0.007 |
